# Engineering of nickel, cobalt oxides and nickel/cobalt binary oxides by electrodeposition and application as binder free electrodes in supercapacitors

**DOI:** 10.1038/s41598-023-42647-4

**Published:** 2023-09-20

**Authors:** Qaisar Abbas, Hafsa Khurshid, Rahana Yoosuf, Jonathan Lawrence, Bashar A. Issa, Mohammad Ali Abdelkareem, Abdul Ghani Olabi

**Affiliations:** 1 School of Computing, Engineering and Physical Sciences, Institute of Thin Films, Sensors and Imaging, (ITFSI), University of the West of Scotland, Glasgow, PA1 2BE UK; 2 Department of Applied Physics and Astronomy, University of Sharjah, Sharjah, 27272 UAE; 3 Thayer School of Engineering, Dartmouth College, Hanover, NH 03756 USA; 4 Department of Medical Diagnostic Imaging, University of Sharjah, Sharjah, UAE; 5 Sustainable Energy & Power Systems Research Centre, RISE, University of Sharjah, P.O. Box 27272, Sharjah, UAE; 6 Department of Biomedical Engineering, Faculty of Engineering and Natural Sciences, Istinye University, Istanbul, 34010 Turkey

**Keywords:** Engineering, Materials science, Physics

## Abstract

Cobalt oxide, nickel oxide and cobalt/nickel binary oxides were synthesised by electrodeposition. To fine tune composition of CoNi alloys, growth parameters including voltage, electrolyte pH/concentration and deposition time were varied. These produced nanomaterials were used as binder free electrodes in supercapacitor cells and tested using three electrode setup in 2 MKOH aqueous electrolyte. Cyclic voltammetry and galvanostatic charge/discharge were used at different scan rates (5–100 mV/s) and current densities (1–10 A/g) respectively to investigate the capacitive behaviour and measure the capacitance of active material. Electrochemical impedance spectroscopy was used to analyse the resistive/conductive behaviours of these electrodes in frequency range of 100 kHz to 0.01 Hz at applied voltage of 10 mV. Binary oxide electrode displayed superior electrochemical performance with the specific capacitance of 176 F/g at current density of 1 A/g. This hybrid electrode also displayed capacitance retention of over 83% after 5000 charge/discharge cycles. Cell displayed low solution resistance of 0.35 Ω along with good conductivity. The proposed facile approach to synthesise binder free blended metal electrodes can result in enhanced redox activity of pseudocapacitive materials. Consequently, fine tuning of these materials by controlling the cobalt and nickel contents can assist in broadening their applications in electrochemical energy storage in general and in supercapacitors in particular.

## Introduction

Contribution of energy generated through renewable and sustainable sources such a wind, tidal and solar is growing significantly due to the repaid growth in demand and impact of fossil fuel-based energy production on the environment^1^. However, these renewable and sustainable sources of energy are intermittent in nature and require appropriate energy storage systems. Energy storage devices such as rechargeable batteries and supercapacitors (SCs) are becoming extremely widespread to address the intermittency of these renewable and sustainable sources of energy. SCs have attracted immense research interest recently since these devices can bridge the energy and power densities gap between rechargeable batteries and traditional electrostatic capacitors^2^. Furthermore, characteristics such as exceptionally high-power densities, outstanding stabilities and excellent efficiencies make SCs highly desirable electrical energy storage devices. Based on the charge storage mechanism, SCs can be divided into two main categories i.e., electric double layer capacitors (EDLCs) and pseudocapacitors (PCs). In EDLCs, charge is stored on the electrode/electrolyte interface through physical ionic adsorption. Capacitance in EDLCs depends on the active material’s accessible surface area and the distance between centres of electrolyte ions and atoms of the active material. Carbon based materials such as activated carbon, graphene, carbon nanotubes and polymeric carbon are the most commonly used electrode materials in EDLCs^3,4^. Whereas pseudo-capacitive energy storage is based on fast and fully reversible Faradic reaction involving electronic transfer at or near the surface of electrodes^5,6^. Typical electrodes of PCs are based on transition metal oxides, hydro oxides or conducting polymers. Electrode is a key component of a supercapacitor cell since it can have a significant impact on its performance. Therefore, selection of suitable electrode material is of utmost importance. The advantages in using metal oxides based nanostructured materials for supercapacitor applications are generally attributed to their large surface area^7^, exceptional mechanical, and electrical properties by virtue of their confined dimensions, short diffusion path lengths and high theoretical capacitance^8,9^. Different nano-structures with particular architectures including nanorods, nanowires, nanosheets, nano-cubes and nanospheres have been intensively utilised and widely reported in literature as advanced electrode active materials for SCs^10^.

RuO_2_ is considered the most promising electrode material when compared with other transition metal oxides since it has superior theoretical capacitance and excellent electrical conductivity. However, toxicity and elevated cost associated with RuO_2_ makes it less desirable for its application as an efficient active material for SCs. Consequently, wider commercialisation and real word applications of SCs require nanomaterials based on traditional metal oxides/hydro oxides which are inexpensive, and less contaminating. Different transition metal oxides such as MnO_2_, NiO and Co_3_O_4_ have been investigated intensively with an aim to deliver superior capacitive performance in a cost effective and environmentally friendly manner. Nickel and cobalt oxides are of enormous interest as pseudocapacitive electrodes due to their high specific capacitance, energy densities, thermal/chemical stabilities, ease of fabrication, as well as low-cost and environmental benignity^11,12^. NiO, like Co_3_O_4_ has been widely evaluated as an electrode material in SCs due to its cost-effectiveness, natural abundance, and good specific capacitance. NiO is one of the most research metal oxide for SC’s applications, however, inferior electrical conductivity and poor reversibility are some of the key disadvantages associated with the wider adoption of NiO. Recent literature suggests that transition metal oxide based composite materials exhibit better performance than a single or individual material for supercapacitor applications thanks to their enhanced chemical activity, stability and electrical conductivity^13^. Therefore, coupling NiO with other materials such graphene, activated carbon and metals oxides such as Co_3_O_4_ has resulted in improved capacitive performance where each component complements other and actively contribute towards overall performance enhancement^14–18^. Surface morphologies and deposition methods can also have a considerable impact on the electrochemical performance of SC cell since these can greatly impact charging and discharging processes^19^. Furthermore, use of organic binders during the production of electrodes for SC cells can have a negative impact on the performance of these device, as binders are usually comprised of insulating materials which can result in increased material resistance due to immobility in binder and stocking effect in electrode materials^20^. While ‘‘binder free electrodes’’ are a novel approach to overcome the limitation posed by these traditional binders. With the exclusion of binders, researchers are seeking to enhance the performance of electrochemical energy storage devices i.e., supercapacitors while making these devices more environmentally friendly. Binder free electrodes are typically fabricated through the direct growth of active material on current collector’s surfaces or by using self-standing films. Electrolysis is a promising method to deposit metal nanostructures directly onto current collector’s surfaces such as carbon cloth (CC), followed by directly using these as binder-free electrodes for SC’s applications. Selection of CC as current collector in this particular study is due to added benefits such as the 3D porous network structure of CC to adsorb more ionic electrolyte together with the outstanding electrical conductivity^21^. The establishment of electrolytic cell with highly conductive CC as working electrode, the nanostructures can be directly deposited on CC easily by electrolysis. The deposition without organic additive is advantageous to bring the best of the synergetic effects of metal and highly conductive carbon. This study distinguishes itself from other works on three key merits which are successfully incorporated in a single study, (1) effective synthesis of Co, Ni monometallic oxides and CoNi binary oxides with precisely tuned composition using facile electrodeposition method, (2) production of binder free transition metal oxides-based electrodes through electrodeposition eliminating the requirement of non-conducting binders, (3) manufacturing of very thin and light weight electrodes where lightweight/thinness of electrodes can be highly beneficial for the superior capacitance of a SC cell. Since, electrode material only on/near surface actively contributes towards electrochemical performance of a SC whereas light weight electrodes can have superior capacitance performance as given by Eq. (1). Nickel (Ni), Cobalt (Co), and Cobalt/nickel binary oxides (CoNi) were directly synthesised on CC. Electrodes comprised of directly deposited active material on CC where CC was used as both the current collector and substrate were investigated for their electrochemical performance. Specific capacitance of 176 F/g at current density of 1 A/g was attained for CoNi binary oxide with optimized ratios of 81.6% and 18.4% for Co and Ni respectively which was found to be much superior then the capacitance of monometallic oxide-based electrodes. Furthermore, this supercapacitor device using CoNi as an active material displayed superior energy density at high power density coupled with excellent cycling stability.

## Experimental

### Synthesis of cobalt (Co), nickel (Ni), and cobalt/nickel (CoNi) alloy

**Figure 1:**
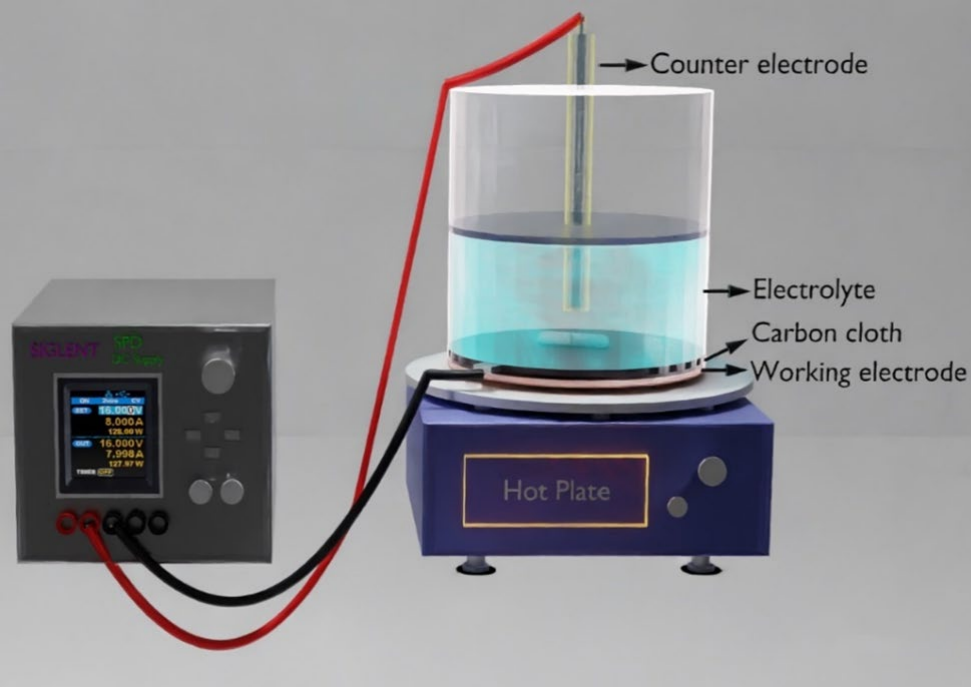
Schematic illustration of experimental setup used for electrodeposition of Co, Ni and CoNi nanomaterials.

In this study, we use a simple and facile two electrode system to deposit the Co, Ni and CoNi composite on carbon cloth by electrochemical deposition method. The standard two-electrode cell for electrochemical deposition consists of a working electrode and a counter electrode which are connected to a DC power supply. A number of different materials can serve as working and counter electrodes. In the current synthesis procedure, the carbon cloth was used as the working electrode and graphite as the counter electrode in the two-electrode system. The carbon cloth is connected to the DC powers supply via a copper plate. The deposition parameters such as the electrode area, voltage, temperature, and electrolyte concentration were fixed as per the previously optimized procedures for relevant metals. A schematic diagram of the electrochemical cell connected to DC power supply is shown in Fig. 1.

To obtain nanostructure coated carbon cloths, the metal nanostructures were deposited within the channels of the carbon cloth following the optimized fabrication conditions and protocols^22,23^ which involved electrochemical deposition in the two-electrode cell with a bath volume of 20 ml. All depositions were performed at room temperature. A magnetic stirrer was used in all experiments. The exposed apparent area of the working electrode was 1.76 cm^2^. To ensure a uniform electric field at the working electrode (CC), the counter electrode was kept at 3 cm from CC. All reagents used were of analytical grade and used without any chemical alteration. The precursor for the deposition of cobalt consisted of 0.2 M CoSO_4_⋅7H_2_O and 0.5 M of H_3_BO_3_. pH was adjusted to 3.3 by adding dil. H_2_SO_4_. The voltage of deposition was 4 V. Nickel nanostructures were deposited from a solution of 0.2 M NiSO_4_⋅6H_2_O and 0.5 M of H_3_BO_3_, with pH 3.8 under constant voltage of 4 V. Nanocrystalline Co–Ni alloys were electrodeposited on CC from an electrolyte solution which consisted of CoSO_4_⋅7H_2_O, NiSO_4_⋅6H_2_O and H_3_BO_3_. Growth parameters including voltage, electrolyte pH, duration of deposition, electrolyte concentration was changed in order to alter alloys composition. The optimum electrochemical characteristics were obtained for Co–Ni alloy prepared with a deposition voltage of 3.5 V, and deposition duration of 1 h from an electrolyte solution contained 0.1 M of CoSO_4_⋅7H_2_O, 0.1 M of NiSO_4_⋅6H_2_O and 0.1 M H_3_BO_3_. The pH of the solution was 3.61.

### Material characterization of metals and bimetallic alloys deposited on CC

The metals and their alloys deposited on CC were subjected to X-ray diffraction (XRD), Scanning Electron Microscopy (SEM), Energy Dispersive X-ray Spectroscopy (EDX), and Elemental Mapping. The morphology and microstructure were observed by SEM (FESEM thermoscientific Apreo C), XRD (Bruker D8 ADVANCE diffractometer) with CuKα radiation and by X-ray Photoelectron Spectroscopy (XPS, Nexsa G2, Thermoscientific, U.K equipped with mono-chromatised Al-Kα radiation (1486.6 eV)). The chemical composition of the nanostructures was analyzed using EDX and elemental mapping (Tescan Vega 3, Tescan Analytics).

## Results and discussions

**Figure 2:**
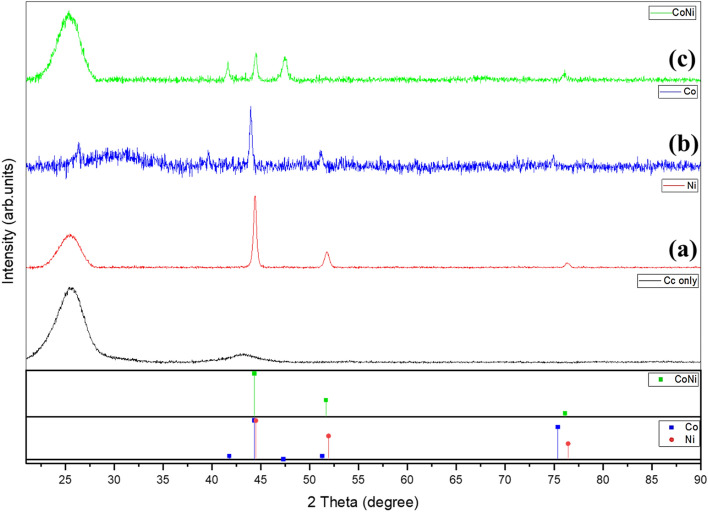
XRD pattern of as-deposited Ni nanostructure on CC, Co nanostructure on CC, CoNi alloy nanostructure on CC with their relevant JCPDS files.

The X-ray crystallographic analysis was used to study the structural analysis of Ni and Co and CoNi alloy as shown in Fig. 2. The XRD patterns of all samples showed a major carbon peak from CC. XRD of the as-deposited Ni on CC (Fig. 2a) shows the 2θ values for the planes (111), (200) and (220) at 44.44°, 51.89° and 76.35° respectively. The XRD pattern indicates that the deposited Ni is highly crystalline in nature and that they crystallize in face centred cubic (fcc) phase with a preferential growth along (111) (JCPDS file no 04850). No distinct diffraction peak other than those from fcc-Ni is found in the sample. The formation of hexagonal close packed (hcp) phase of Cobalt is shown in Fig. 2b.

The XRD pattern of the deposited Co–Ni alloy is shown in Fig. 2c. As depicted in the figure, a hexagonal close-packed (hcp) crystal structure with distinct hkl planes (100) (002), (101 and (110) (ICDD PDF Card 04-004-8488) were observed at 2θ values 41.6°, 44.4°, 47.4° and 76° respectively which can be attributed to the higher cobalt content as confirmed from EDX analysis. Similar results were reported for previous research on hcp structured CoNi alloy with high cobalt content^24–26^. The elemental compositions from EDX were found to be 81.6 (wt%) of Co and 18.4 wt% of Ni. If the layer of the deposited film is thick enough, the X-rays beam cannot penetrate all the way through the film which weakens the signals from the underlying material. That explains a weaker CC peak in Co sample.

**Figure 3:**
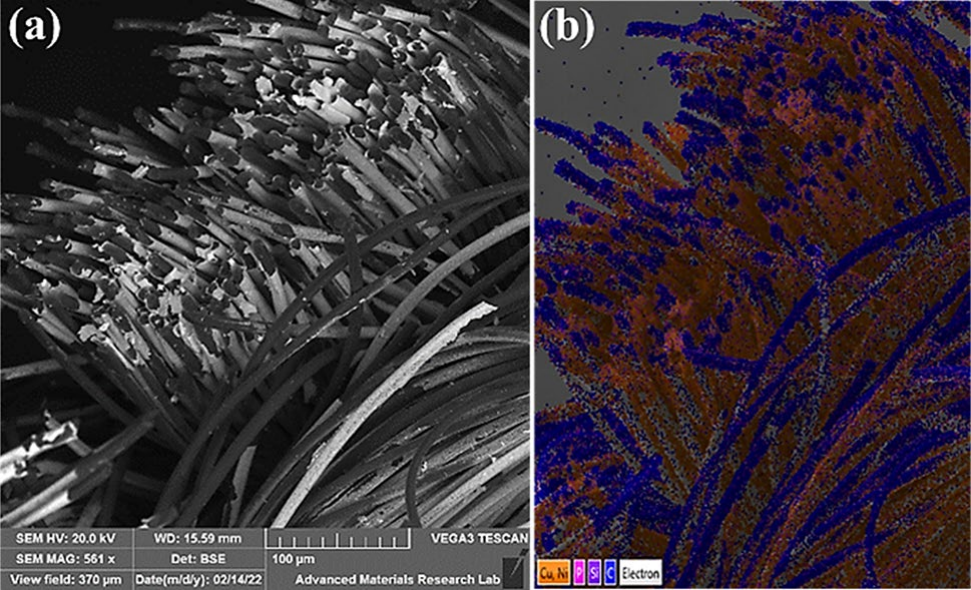
Nickel on carbon cloth **(a)** SEM micrograph, **(b)** EDX layered image.

**Figure 4:**
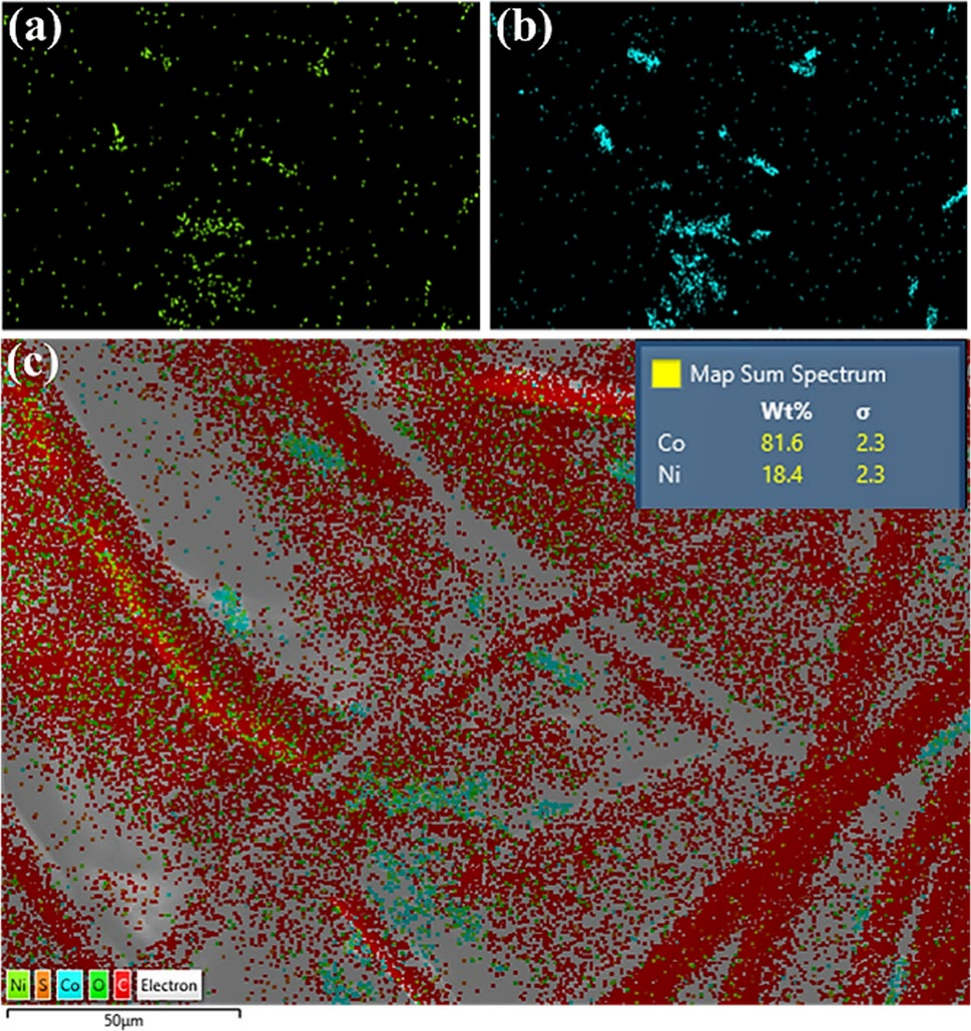
Area mapping of Ni, Co, and CoNi alloy at magnification of 50 µm. The composition of Co and Ni from EDX analysis is given in inset.

The typical morphology of the deposited nanostructure in CC is shown in Fig. 3 whereas Fig. 4 is the EDX analysis showing composition of Co and Ni in CoNi alloy. The alloy formation of Co and Ni can be confirmed from the colour distribution also.

**Figure 5:**
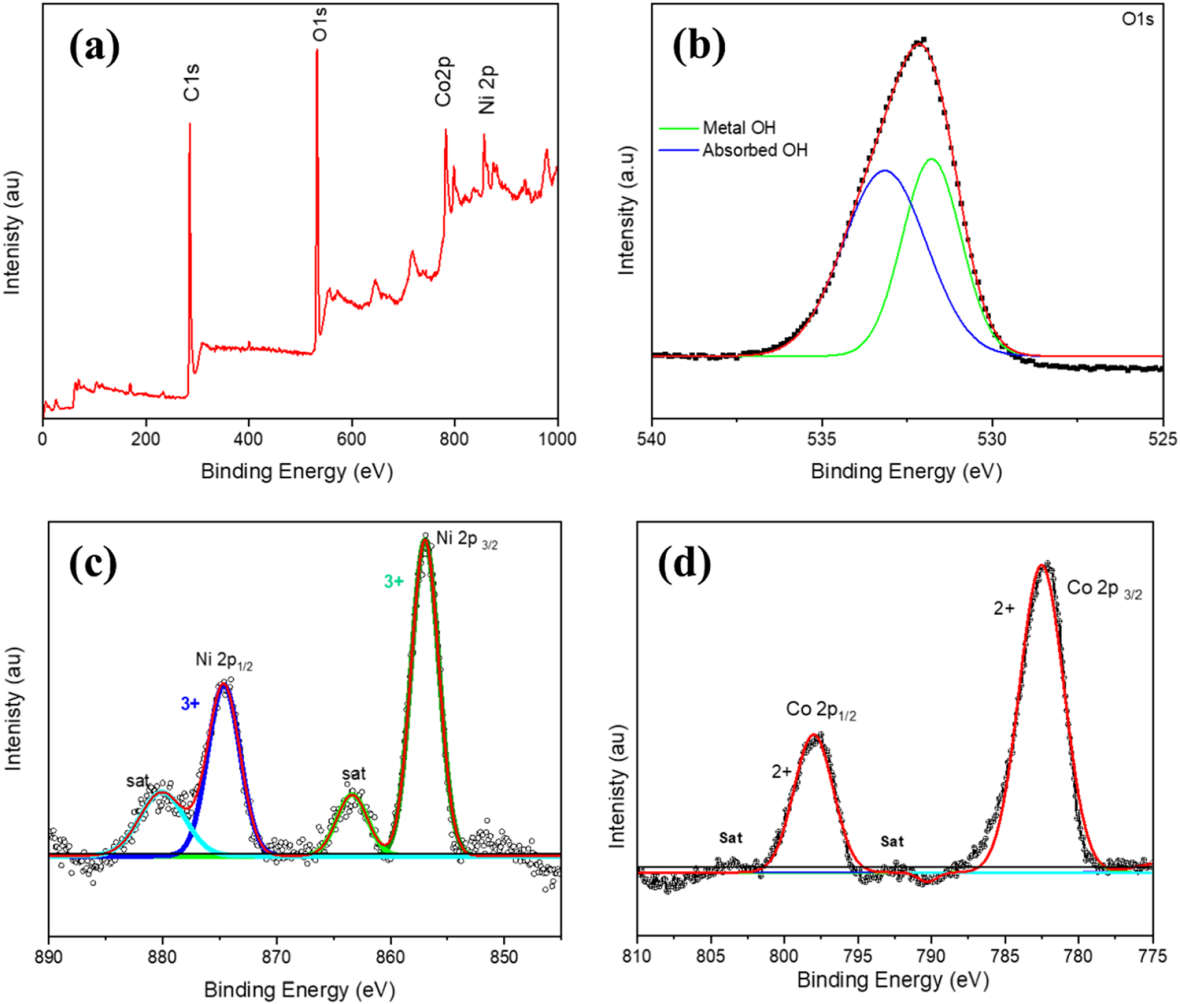
**(a)** XPS survey scan for CoNi composites deposited on carbon cloth, high resolution XPS spectrum of **(b)** O1s, **(c)** Ni2p and **(d)** Co2p. The black scattered line is the original signal, and the red curve is the result of the curve fit.

The more detailed elemental composition and electron structure of the as-prepared CoNi hybrid deposited on CC was further analysed by XPS where the working pressure in the spectrometer chamber was ~ 10–9 mbar. Flood gun was used for charge compensation and spot size was 400 µm spectra, broad XPS spectra is displayed in Fig. 5a.

Figure 5a also shows that the surface has a composition of Ni, Co, and O. The peaks 2p_1/2_ and 2p_3/2_ resulting from the spin–orbit splitting of Ni_2_p and Co_2_p is shown in Fig. 5b,c respectively. A comprehensive analysis of the oxidation state of the elements were performed employing a Gaussian fitting method. Figure 5b shows the fitting peaks at 856.23 eV and 874.20 eV corresponding to Ni ^3+^^23–25^, with the satellite peaks at 863.37 eV and 880.06 eV. The Co 2p_3/2_ and Co 2p_1/2_ peak at 782.09 and 797.12 eV shown in Fig. 5c corresponds to Co^2+^ whereas the peaks at 785.4 eV and 798 eV are ascribed to satellite peaks. The typical metal–oxygen bonds are at the O1 peak at 529.1 eV^26^ and the peak at 531.2 eV corresponds to OH group^27^. The peak of metal–oxygen can’t be observed in the current scan and the O1 peak located at 531.9 eV can be assigned to OH group. The peak at 533.1 eV might be due to defects, contaminants, or multiplicity of physic-/chemisorbed water at/within the interface of the material^28–30^.

### Electrochemical characterisations

The electrochemical performance of active material was assessed using the Biologic VSP-200 in a typical three-electrode setup. For the three-electrode configuration, prepared materials deposited on CC were directly used as working electrodes (1 cm^2^), the platinum wire was used as a counter electrode, Hg/HgO was used as a reference electrode and 2 MKOH aqueous solution as an electrolyte. The electrochemical behaviour and capacitance were evaluated using cyclic voltammetry (CV) and galvanostatic charge–discharge (GCD) measurements, recorded at different scan rates and current densities respectively. The electrochemical impedance spectroscopy (EIS) analysis was performed at frequency ranges from 100 kHz to 0.01 Hz at applied voltage of 10 mV to understanding the conductive/resistive behaviour of active samples. The specific capacitance (Q_s_) was calculated from the discharge curve using the following formulae:

1 $${Q}_{S}=\frac{I\times \Delta \mathrm{t}}{m\times \Delta V},$$

where I (A) represents the discharge current density, ∆t (s) represents the discharge time, m (g) is the mass of active material, ∆V is the voltage window.

Specific energy (E, Wh/kg), and specific power (P, W/kg) of the supercapacitor were calculated using Eqs. (2) and (3) respectively.

2 $$E=\frac{0.5\times {C}_{sc}\times \Delta {V}^{2}}{3.6},$$

3 $$P=\frac{E\times 3600}{\Delta t}.$$

**Figure 6:**
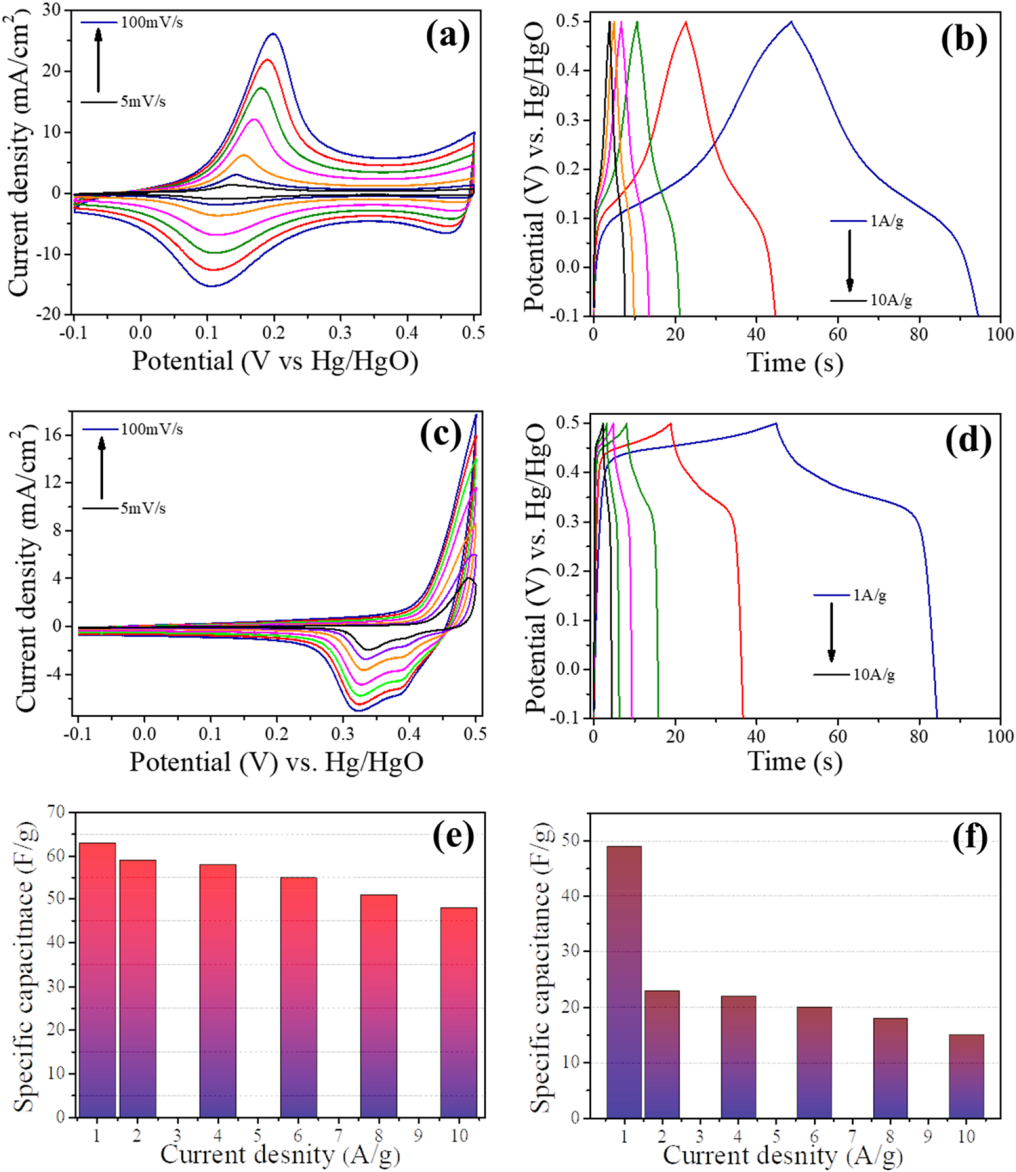
**(a)** CV profile of Co sample at the scan rates of 5–100 mV/s. **(b)** GCD curve of Co sample at current densities of 1–10 A/g. **(c)** CV profile of Ni sample at the scan rates of 5–100 mV/s. **(d)** GCD curve of Ni sample at current densities of 1–10 A/g, **(e)** current density vs specific capacitance plot of Co sample and **(f)** current density vs specific capacitance plot of Ni electrode.

Figure 6a shows the cyclic voltammograms (CV) of cobalt oxide-based electrode at different scan rates ranging from 5 to 100 mV/s. Well defined redox peaks during both charging and discharging phases are originating from interaction of hydroxyl ions with the relevant active material. Higher scan rate results in increased redox peak intensity and shift these peaks towards slightly higher potentials signifying the fast redox reaction taking place at the electrode/electrolyte interface. Slight drop in capacitance at higher scan rates was observed as shown in Fig. 6e, which was anticipated due to limited ionic diffusion, restricted ion adsorption and poorer charge transfer at higher rates^31^. However, the CV profile remained symmetric without any significant deformation indicating that Co based active material displayed an excellent rate capability.

Charge storage mechanism and presence of redox peaks in Fig. 6a of cobalt oxide based pseudocapacitive material in 2 MKOH electrolyte can be described by Eqs. (4) and (5)^32^.

4 $${\mathrm{Co}}_{3}{\mathrm{O}}_{4}+{\mathrm{OH}}^{-}+ {\mathrm{H}}_{2}\mathrm{O }\leftrightarrows 3\mathrm{CoOOH}+ {\mathrm{e}}^{-},$$

5 $$\mathrm{CoOOH}+\mathrm{ O}{\mathrm{H}}^{-}={\mathrm{CoO}}_{2}+ {\mathrm{H}}_{2}\mathrm{O}+{\mathrm{e}}^{-}.$$

Figure 6b shows the highly symmetric GCD profile of Co electrodes in potential window of − 0.1 to 0.5 V at current densities of 1, 2, 4, 6, 8 and 10 A/g indicating excellent reversibility during charge/discharge processes, this behaviour is in excellent agreement with CV profiles^33^.

CV profiles of Ni based active material deposited on CC in the potential range of − 0.1 to 0.5 V at scan rates of 5 to 100 mV/s are shown in Fig. 6c. Two distinctive oxidation/reduction peaks can be observed of Ni electrode in 2 MKOH electrolyte where oxidation of NiO to NiOOH and reduction of NiOOH to NiO occurs which can be represented by Eq. (6)^34^.

6 $$\mathrm{NiO}+{\mathrm{OH}}^{-} \leftrightarrows \mathrm{ NiOOH}+ {\mathrm{e}}^{-}.$$

It was also observed that oxidation peaks shifted to higher potentials while reduction peaks moved to lower potentials with an increase in the scan rates. This can be credited to the limitation of ions diffusion and movement at higher scan rate during the redox reaction. Furthermore, it can be assumed that this phenomenon can be associated with the higher series resistance of NiO^35^.

It can be seen from Fig. 6d, GCD profile of Ni based electrodes in the potential range of − 0.1 to 0.5 V that a voltage plateau appears during charge cycle around 0.42 V and during discharge voltage plateau is from 0.4 to 0.32 V which confirms the occurrence of a pseudocapacitive reaction in the charge/discharge processes. Moreover, charge discharge time are similar which indicates good reversibility of redox reaction. It can also be observed from Fig. 6e,f that the drop in capacitance of Ni electrode is much higher when compared with Co electrode at higher current densities which is mainly due to the slower redox kinetics in Ni because of higher series resistance (which will be discussed in detail in later section). Higher series resistance can result in low transfer rate of electrons and low diffusion rate of ions. Higher resistance is also evident from the comparison presented in Fig. 9d,e of the electrochemical impedance spectroscopy (EIS) analysis for Co, Ni and CoNi electrodes.

**Figure 7:**
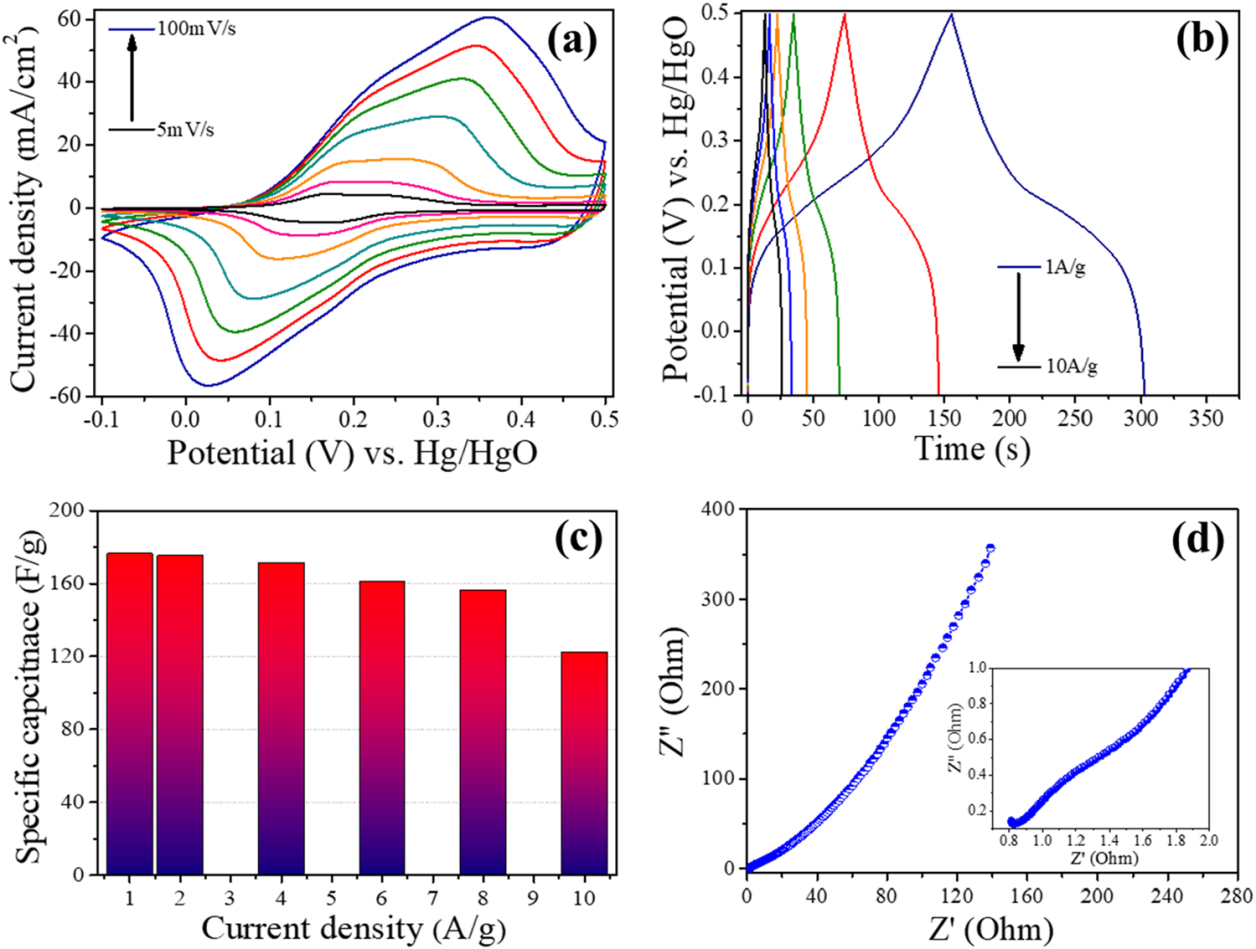
**(a)** CV profile of CoNi sample at current densities of 5–100 mV/s, **(b)** GCD curve of CoNi sample at scan rates of 1–10 A/g, **(c)** current density vs specific capacitance plot. **(d)** Nyquist plot of CoNi based sample in frequency range of 100 kHz to 0.01 Hz.

Similarly, CoNi alloy was also investigated under analogues parameters using CV, GCD and EIS techniques. CV profiles of CoNi alloy at the scan rates of 5 to 100 mV/s in the potential range − 0.1 to 0.5 V are shown in Fig. 7a. Two strong redox peaks can be witnessed indicating typical pseudocapacitive behaviour of CoNi based active material. These two peaks can be explained on the basis of electrochemical phenomenon taking place in aqueous media which is given by Eqs. (7) and (8).

7 $${\mathrm{NiCo}}_{2}{\mathrm{O}}_{4}+\mathrm{O }{\mathrm{H}}^{-}+ {\mathrm{H}}_{2}\mathrm{O}=\mathrm{NiOOH}+2\mathrm{CoOOH}+ {\mathrm{e}}^{-},$$

8 $$\mathrm{CoOOH}+{\mathrm{OH}}^{-}={\mathrm{CoO}}_{2}+ {\mathrm{H}}_{2}\mathrm{O}+ {\mathrm{e}}^{-}.$$

Pair of these redox peaks around 0.1 and 0.35 V are associated with the reactions Ni^2+^/Ni^3+^, Co^2+^/Co^3+^ and Co^3+^/Co^4+^ associated with OH^−^ to form NiOOH, CoOOH, and CoO_2_, respectively^36^. From Figs. 6a,c and 7a it can be seen that the reduction potential of CoNi binary compound is lower than CO and Ni individually which can be due to its high valence ions^37^. Also, at higher scan rates peak potentials shifted and peaks separation increased which is indictive of quasi-reversible nature of electrode materials which is the typical behaviour of pseudocapacitive material^38^.

Figure 7b shows the GCD cycles of CoNi electrode at various current densities ranging from 1 to 10 A/g in the potential range of − 0.1 to 0.5 V. All GCD profiles displayed clear potential plateau regions with battery type characteristics indicating outstanding pseudocapacitive behaviour^39^. Furthermore, all GCD profiles also exhibited near symmetric charge–discharge patterns and extremely reversible charge–discharge properties, revealing the superior reversibility of redox reaction along with outstanding capacitive properties. Specific capacitance was calculated from the discharge components of GCD curves and calculated capacitances at different current densities is shown in Fig. 7c. It was observed that similar to Co and Ni samples, drop in capacitance was observed for CoNi when current density was increased this can be due to the diffusion and penetration of electrolyte solution’s ions at much higher current densities^40–42^. However, it can be witnessed that CoNi based active material displayed excellent capacity retention at higher current densities where specific capacitance value dropped moderately from around 176 F/g at current density of 1 A/g to around 122 F/g when current density was increased by around ten times to 10 A/g. It can also be observed that compared with other samples i.e., Co (63 to 45 F/g) and Ni (49 to 25 F/g), drop in capacitance of CoNi sample when current densities are increased from 1 to 10 A/g is substantially lower which can be due to the fact that binary oxides with CoNi being a typical example can afford a richer variety of redox reactions (contribution made by both Ni^2+^ and Co^3+^ ions) and possess much superior electrical conductivities compared with monometallic oxide materials^43^. EIS technique was utilized to analyse the electrical resistivity of CoNi based electrode material and Nyquist plot of this binary compound-based electrode is shown in Fig. 7d. Solution resistance (Rs) which includes interfacial contact resistance between active materials and current collector, the inherent resistance of active material and ionic resistance associated with electrolyte solution is represented by the intercept of Nyquist plot at the real axis (x-axis) in high frequency region^44,45^. CoNi based sample manifested small x-axis intercept in high frequency region (inset in Fig. 7d) displaying low internal resistance which is in line with the results of other electrochemical performance characteristics.

**Figure 8:**
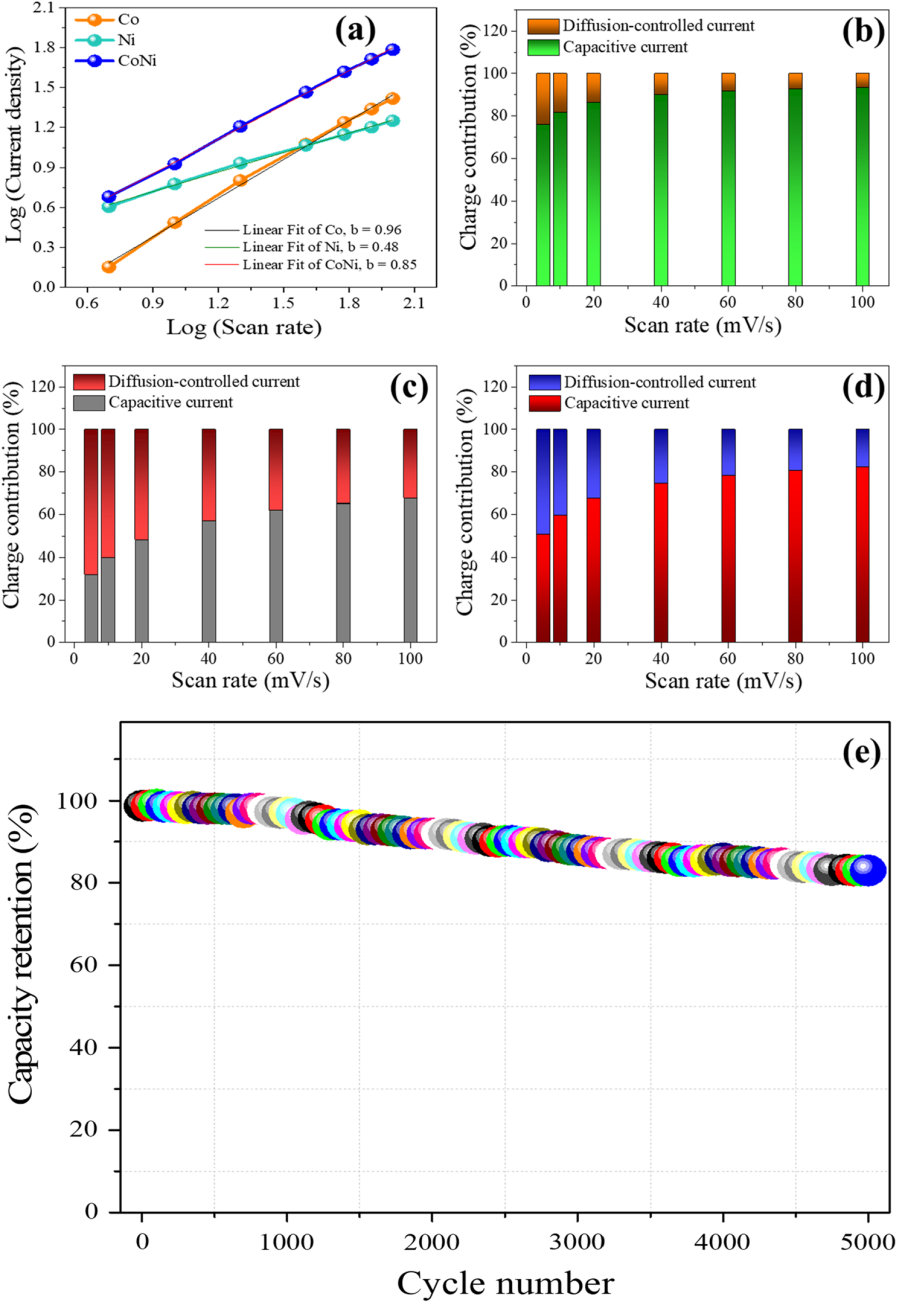
**(a)** Plot of log (current density) versus log (scan rate) of Co, Ni and CoNi electrodes, **(b)** the contribution ratio of capacitive and diffusion-controlled currents of Co electrode**, (c)** the contribution ratio of capacitive and diffusion-controlled currents of Ni sample, **(d)** the contribution ratio of capacitive and diffusion-controlled currents of CoNi sample. **(e)** Cyclic stability plot of CoNi after 5000 charge/discharge cycle.

To gain enhanced insight of the overall electrochemical performance and charge storage dynamics of Co, Ni and CoNi electrodes, the power law $(I=a.vb,$ where *‘a’* and *‘b’* are adjustable) was applied where relationship between current (i) and scan rates (v) was examined^46^. ‘*b*’ value is the limiting parameter of the power law equation, *b* = 1 symbolises surface controlled capacitive process whereas *b* = 0.5 signifies the diffusion-controlled contribution. Frome the CV profiles of Co, Ni samples from Fig. 6a,c and CV profile of CoNi samples displayed in Fig. 7a at scan rates 5 to 100 mV/s, the fitted b-values for Co, Ni and CoNi are 0.96, 0.48 and 0.85 respectively as shown in Fig. 8a. This indicates the contributions were made from both diffusion-controlled as well as capacitive-controlled electrochemical processes. The b-values for Co and CoNi samples are superior and are close to *‘1’* when compared with Ni sample which implies that the capacitive process is dominated by capacitive behaviour based on surface redox reactions. Furthermore, the charge storage contribution ratios of Co, Ni and CoNi electrodes is measured by evaluating individual charge storage contributions elements conferring to the modified power law,

9 $$i={k}_{1}v+ {k}_{2}{v}^{1/2}.$$

Revised to

10 $$i/{v}^{1/2 }= {k}_{1}{v}^{1/2}+ {k}_{2},$$

where, *k*1 and *k*2 are the variables for capacitive and diffusion-controlled processes, respectively^47^. It can be observed from Fig. 8b–d that calculated percentage of capacitive charge storage contribution is significantly higher for Co monometallic and CoNi composite sample. This outstanding contribution of capacitive contribution of cobalt oxide and CoNi binary oxide is due to superior capacitive characteristics of Co and the complementary contribution of individual metal oxides for CoNi sample. It can also be observed from Fig. 8b–d that the capacitive contribution is the dominant component which increases with increase in scan rates implying that most of the total capacitance is originating from surface redox reactions and large surface polarisation. Therefore, it can be concluded that the ion kinetics in Co and CoNi deposited electrodes contributes predominately from pseudocapacitance (surface redox reactions) when compared with diffusion-controlled capacitance initiating from intercalation/deintercalation of electrolytic ions.

Robustness of CoNi based active material was further examined by cycling through 5,000 charge–discharge cycles at the current density of 10 A/g to evaluate this electrode material for real-world applications. Cell displayed slight degradation with the retention of over 83% of the initial capacitance after 5000 cycles as shown in Fig. 8e. This demonstrates that CoNi based binary compound can be a promising electrode material for energy storage applications in supercapacitors. Supercapacitors cell also delivered the energy density of 29 Wh/kg at the power density of 733 W/kg and retained energy density of 23 Wh/kg at an ultra-high-power density of 7320 W/kg. These energy/power density results demonstrate that supercapacitor cell based on CoNi composites can deliver much higher power without losing energy storage capability substantially. Since there was a slight drop in energy density whereas the power density was increased nearly tenfolds.

**Figure 9:**
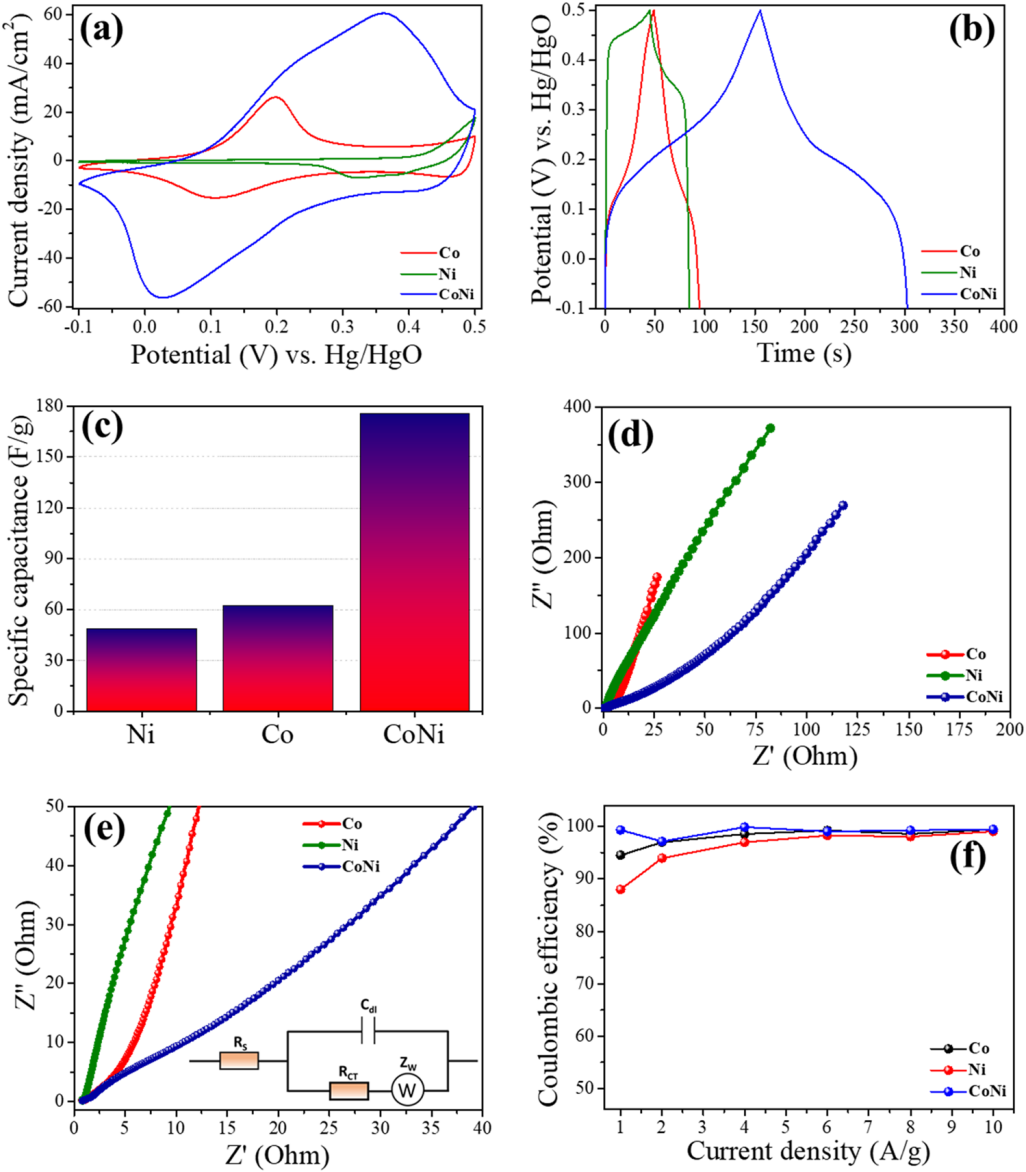
Comparison of **(a)** CV profiles of Co, Ni and CoNi samples at scan rate of 100 mV/s, **(b)** GCD curves of Co, Ni and CoNi samples at current density of 1 A/g, **(c)** capacitive performance of Co, Ni and CoNi samples, **(d)** EIS profile of Co, Ni and CoNi samples in frequency range of 100 kHz to 0.01 Hz, **(e)** magnified Nyquist plot along with equivalent circuit for all the samples and **(f)** comparison plot of Coulombic efficiency for Co, Ni and CoNi electrodes.

Performance comparison for individual samples was conducted in order to understand electrochemical behaviour of each type of active material. Figure 9a displays the CV profiles comparison of Co, Ni and CoNi samples where it can be observed that the area underneath CoNi CV curve is much larger when compared with other two monoatomic samples i.e., Co and Ni. Therefore, it can be implied that the redox active sites are much larger in numbers for CoNi sample when compared with either Co or Ni which provides the basis for this sample to display much superior capacitive performance. Therefore, this can be concluded that this binary compound is a more suitable active material for supercapacitors with much higher specific capacitance when compared with its parent monometallic samples. A comparison of GCD profiles of Co and Ni based monometallic samples and CoNi based binary compounds is also shown in Fig. 9b. These results are in good agreement with the CV results as discharge time for CoNi binary compound is more than three times to those of Co and Ni samples. This increased discharge time can be attributed to synergetic effect of Co, Ni species and electrochemically active CoNi in binary compound of CoNi. The calculated specific capacitances using Eq. (1) are 176, 49 and 63 F/g for CoNi, Ni and Co respectively as shown in Fig. 9c. The electrochemical performance of all three samples can be linked closely with the respective GCD and CV profiles of each sample meanwhile confirming the superior performance of CoNi sample.

EIS was used to analyse resistive/capacitive behaviour of these samples. The Nyquist plots for Co, Ni and CoNi samples are shown in Fig. 9d where the intersects on the real axis correspond to the solution resistance (R_S_) which is the sum of ionic resistance of electrolyte, intrinsic resistance of electrode, and contact resistance at the electrode/electrolyte interface^48^. The diameter of the semicircle in the Nyquist plot corresponds to the charge-transfer resistance (R_CT_) while, in lower frequency region the linear component is associated with Warburg resistance (Z_W_), which is essentially the resistant component of ionic diffusion/transfer in the electrolyte^49^. Randle circuit was used as equivalent circuit and *Z-view Software* was used for EIS plot fitting to obtain R_S_ values where R_S_ values for Co, Ni and CoNi were 0.26 Ω, 0.51 Ω and 0.35 Ω respectively. All sample displayed excellent electrical conductivities with low R_S_ values < 1 Ω where the key contributing factors towards lower R_S_ can be the absence of non-conductive binder and excellent conductivity of carbon cloth. Composite electrode comprising of CoNi with superior capacitive performance has the R_S_ value 0.35 Ω signifying excellent electrical conductivity of hybrid sample. In summary CoNi electrode delivered outstanding charge transfer kinetics demonstrating that the large number of redox reactions can be achieved using this material. Since CoNi has both R_C_ and R_T_ smaller which can result in superior charge transfer kinetics and ultimately large number of redox reactions can be achieved. This can be explained on the basis of band theory as impurity bands can be introduced after mixing these oxide which in turn enhance the electronic conductivity of composites^50^. These superior electrical properties were also confirmed by cyclic stability and rate capability. Low values of x-intercepts for all the samples means lower equivalent resistance and Warburg component at 45° means lower resistive response and excellent capacitive response^48^. Magnified Nyquist plot along with equivalent circuit used for fittings is displayed in Fig. 9e where lower intercept and around 45° angle with real axis of CoNi composite electrode represents excellent conductive and capacitive characteristics making this hybrid a potential electrode active material for applications in supercapacitors. This can also translate into superior specific capacitance as displayed by Fig. 9c. Furthermore, superior electrical conductivity can result in improved rate capability as shown in Figs. 6e,f and 7c where drop in specific capacitance is lower in case of CoNi alloy when compared with Co and Ni samples. Furthermore, Fig. 9f shows the comparison plot of Coulombic efficiency for Co, Ni and CoNi electrodes where all these materials maintained Coulombic efficiency above 99% in cycling test, confirming outstanding reversibility of individual and composite electrodes.

## Conclusions

In summary, we produced Co and Ni oxides, CoNi binary oxides using facile electrochemical deposition technique. By employing this approach Co, Ni and CoNi were directly deposited on highly conductive carbon cloth where carbon cloth was used as both substrate and current collector. During the synthesis of CoNi binary oxides, Co and Ni ratios were fine-tuned by controlling synthesis parameters such as voltage, electrolyte pH, deposition time and electrolyte concentration. The successful deposition of Co, Ni and CoNi was confirmed by number of physical and chemical characterisation techniques. By using this facile technique active material was deposited on carbon cloth and directly used as a binder free electrode in supercapacitor cell. This process provided with an opportunity to produce binder free electrodes with fine-tuned chemical composition. Furthermore, using this process very thin layers of active material can be deposited onto the substrate surface which can be highly useful towards the overall performance enhancement of a SC. Since this can results in the production of light weight and thin electrodes. Reduced weight can result in higher specific capacitance (as given by Eq. 1) also in case of SCs physical/electrochemical reactions take place on/near the surface of active material therefore by producing thin electrodes, electrochemically non-active proportion of an electrode can be reduced considerably. Electrochemical characterisation demonstrated that the binary metal oxides (CoNi) exhibited superior electrochemical characteristics compared with monometallic oxides when these materials were tested using as binder free electrodes in a supercapacitor test cell. Among these samples, CoNi binary oxide with Co and Ni content of 81.6% and 18.4% displayed specific capacitance of 176 F/g much superior to those of 49 and 63 F/g for Ni and Co samples respectively. This composite electrode also exhibited excellent rate capability and long-term stability with the capacity retention of over 83% after 5000 charge/discharge cycles. Electrodeposition based synthesis strategy utilized for the production of transition metal oxides both monometallic and binary oxide can be a valuable addition towards the production of finetuned pseudocapacitive materials and their application as promising electrode materials for the electrochemical energy storage in supercapacitors.

## Data Availability

All data generated or analyzed during this study are included in this published article.
